# Perturbations to Homeostasis in Experimental Models Revealed Innate Pathways Driving Food Allergy

**DOI:** 10.3389/fimmu.2020.603272

**Published:** 2020-12-10

**Authors:** Kelly Bruton, Joshua F. E. Koenig, Allyssa Phelps, Manel Jordana

**Affiliations:** Department of Pathology and Molecular Medicine, McMaster Immunology Research Centre (MIRC), McMaster University, Hamilton ON, Canada

**Keywords:** food allergy, animal modeling, type 2 immunity, Th2, homeostasis

## Abstract

While type 2 immunity has been conventionally viewed as beneficial against helminths, venoms, and poisons, and harmful in allergy, contemporary research has uncovered its critical role in the maintenance of homeostasis. The initiation of a type 2 immune response involves an intricate crosstalk between structural and immune cells. Structural cells react to physical and chemical tissue perturbations by secreting alarmins, which signal the innate immune system to restore homeostasis. This pathway acts autonomously in the context of sterile injury and in the presence of foreign antigen initiates an adaptive Th2 response that is beneficial in the context of venoms, toxins, and helminths, but not food allergens. The investigation of the triggers and mechanisms underlying food allergic sensitization in humans is elusive because sensitization is a silent process. Therefore, the central construct driving food allergy modeling is based on introducing perturbations of tissue homeostasis along with an allergen which will result in an immunological and clinical phenotype that is consistent with that observed in humans. The collective evidence from multiple models has revealed the pre-eminent role of innate cells and molecules in the elicitation of allergic sensitization. We posit that, with the expanding use of technologies capable of producing formidable datasets, models of food allergy will continue to have an indispensable role to delineate mechanisms and establish causal relationships.

## Introduction

Our understanding of the underlying pathogenesis of food allergy and its attendant applications has been largely attained through experimental modeling. In fact, modeling, which has been instrumental to scientific progress in general, is an inescapable approach to decipher complex problems such as a disease. Here, we discuss a broad perspective of type 2 immunity, highlighting its role in homeostasis. Then, we provide a succinct ideology of modeling and examine strategies for food allergy modeling. Lastly, we advance our view on why food allergy modeling will remain an indispensable discovery tool, especially with the continuing development of technologies that facilitate high parameter analyses.

## Pathways of Innate-Driven Type 2 Immunity

Historically, type 2 immunity was conceived as a balancing system for type 1 inflammatory responses and whose primary purpose was to provide defense against helminths (1). Indeed, the symptomatology elicited by type 2 responses, including itching, tearing, intestinal cramps, wheezing, swelling, and, in extreme instances, anaphylaxis, is geared toward containment or expulsion of helminths, poisons, and toxins and are, therefore, beneficial for the host. Antithetically, type 2 immunity promotes harmful responses against food allergens.

Research over the last decade, largely in experimental models, has revolutionized this view ushering in a much richer perspective of both the functions and the engineering of type 2 immunity. With respect to the former, important roles for the cells and molecules of type 2 immunity have been uncovered in a wide range of processes, from tissue repair and metabolism to sensing temperature and chemical imbalances (2). Regarding the engineering, it is clear that the initiation and execution of a type 2 immune response entails an intricate crosstalk between structural and immune cells. Collectively, this research has positioned type 2 immunity as a guardian of homeostasis.

Considerable evidence from multiple contexts has revealed a generic type 2 pathway mediated by innate immune cells and molecules that regulates homeostasis. Structural cells at mucosal sites and the skin, prominently epithelial and endothelial cells, react to tissue perturbations or physiological changes by secreting alarmins, such as IL-33, IL-25, and TSLP (3, 4). These alarmins act on cognate receptors on ILC2s which expand and enact a variety of reparative functions primarily through the secretion of IL-5, IL-13, and amphiregulin (5–7). IL-5 facilitates the accumulation of eosinophils at the tissue site which generally impact homeostasis through their cytokines and granule products (8–12). ILC2s, IL-13, and amphiregulin induce a plethora of developmental and reparative functions. These include but are not limited to promotion of postnatal lung alveolarization (13, 14), stimulating epithelial repair and extracellular matrix production to repair wounds (15–19), regulating muscle cell metabolism and the clearance of necrotic debris during muscular injury and exercise (9, 20, 21), and regulating energy metabolism and the differentiation of beige adipose tissue to increase caloric expenditure and generate heat (22–26). To date, components of this pathway have been implicated in homeostatic maintenance in the skin (18, 19), muscles (9, 20), intestines (27, 28), nervous system (29, 30), liver (8, 31, 32), biliary tract (15), lungs (16), kidney (11), adipose tissue (33), among others. Emergent roles in the initiation and amplification of type 2 immunity are being described for other innate immune cells, such as mast cells and basophils, which produce alarmins and potently secrete other cytokines in some contexts (34, 35). Notably, virtually no adaptive immune involvement has been reported in type 2-mediated homeostasis and, in many cases, occurs in mice without an adaptive immune system.

*Adaptive* type 2 immunity is also activated in the context of significant tissue perturbations. The distinctive rule of engagement is the presence of foreign antigens in the tissue microenvironment. For example, the life cycle of many helminths involves perforating epithelial barriers and implanting into tissues. Most venoms are proteases which cleave proteins critical for tissue integrity and cause necrotic cell death. Some aeroallergens, like house dust mite, have inherent proteolytic activity (36). It follows that type 2 adaptive immunity may have evolved to respond to foreign antigens that are conspicuously present in the local microenvironment of tissue injury. In these alarmin-rich contexts, epithelial cell-derived cytokines directly act through cognate receptors on dendritic cells to condition the expression of the costimulatory molecule OX40L and downregulate IL-12 expression (37, 38). These dendritic cells also capture local antigen and migrate to secondary lymphoid organs where they engage naïve CD4^+^ T cells. OX40L is critical to induce IL-4 secretion from CD4^+^ T cells, which is thought to act in an autocrine manner to upregulate STAT6 and GATA3 resulting in Th2 differentiation (39–41). Some T follicular helper cells acquire a Th2-like phenotype required for the differentiation of IgE-secreting cells (42). Adaptive type 2 immunity is beneficial for the host in the aforementioned contexts; IgE is reported to provide resistance to venoms (43) and Th2 cells and IgE facilitate expeditious clearance of parasites (44–46).

**Figure 1:**
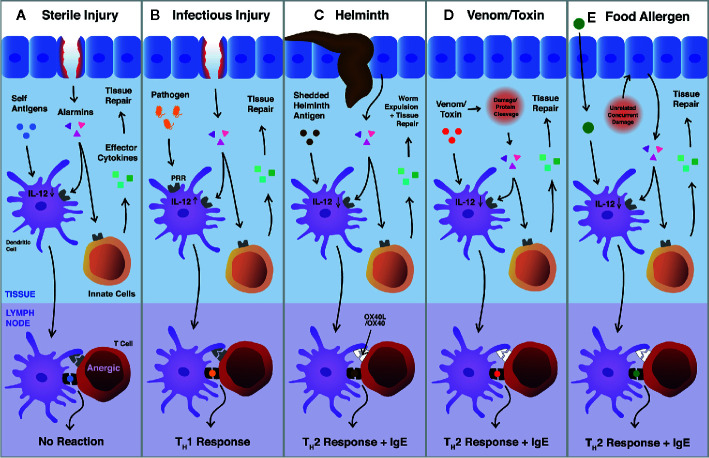
Pathways of Type 2 immune activation *via* tissue perturbations. **(A–D)** Epithelial cells release alarmins (e.g., IL-33, IL-25, and TSLP) in response to tissue damage. Alarmins signal through cognate receptors on innate immune cells, resulting in proliferation and the release of effector cytokines (e.g., IL-5, IL-13, and amphiregulin). These cytokines directly influence tissue repair (e.g., stimulating re-epithelization and extracellular matrix production) or cause the recruitment of other cells (e.g., eosinophils) which aid in tissue repair. Simultaneously, local dendritic cells uptake tissue antigens and respond to alarmin signaling by travelling to the draining lymph nodes and inducing Th2 cell differentiation through OX40-OX40L. **(A)** In the context of sterile injury, only self-antigens are uptaken by dendritic cells and are presented to T cells, which have been limited by central and peripheral tolerance. **(B)** When infectious agents are present, they signal through pattern recognition receptors, upregulating IL-12 in dendritic cells, and initiating Th1 immunity for host defence. **(C, D)** Helminths and venoms, canonical examples of Th2 responses, drive tissue damage by invading tissues or through cleaving proteins, respectively. Antigens that colocalize with damage are presented to T cells and initiate Th2 responses. **(E)** It remains unclear how Th2 immunity is initiated against food allergens. Mouse models require the use of adjuvants (e.g., cholera toxin or tape stripping) to perturb homeostasis, suggesting that food allergic responses are initiated in the presence of damage that is unrelated to the food allergen itself.

We posit that the type 2 innate and adaptive responses are both instigated by the same triggers, perturbations of homeostasis, differing only by the presence or absence of antigen. When tissue homeostasis is disrupted, alarmins signal the innate immune system to initiate reparative functions to regain homeostasis **Figure 1A**). Simultaneously, some of these alarmins condition nearby dendritic cells, which are actively scanning the microenvironment for foreign antigens. Capture of antigens which colocalize with tissue damage are loaded into MHC II and elicit an adaptive type 2 immune response. If a foreign antigen is encountered that signals through pattern recognition receptors (like a bacteria or virus), type 1 immunity is engaged for pathogen control (**Figure 1B**). When no foreign antigens are encountered, DCs internalize and present only self-antigens which usually will not result in adaptive immunity as self-reactive T cells are eliminated or constrained by central and peripheral tolerance (**Figure 1A**). Type 2 immunity is intuitive for helminths, which cause damage when invading tissues and shed antigenic material that is internalized by DCs, thus mounting Th2 and IgE responses (**Figure 1C**) (45). It follows similarly for toxins and some aeroallergens, which cause damage through proteolytic activity and are small enough to be taken up by DCs (**Figure 1D**). However, it falls short when rationalizing immune responses against food allergens, which are highly diverse in structure, the vast majority of which are not immunogenic, and, enigmatically, which are essential for survival.

## Ideology of Disease Modeling

Complexity has driven scientists from all branches to develop models, these understood as the functional simplification of an intractable reality. The development of a scientific model is based on the prevailing interpretation of the available information at a given time. Therefore, models are not static but evolve as we enhance our knowledge of a process. A biomedical model is a surrogate for a human being, or a human biological system. Its primary purpose is to understand how the human body works, from genotype to phenotype. Within biomedical models, animal models provide precise genetic and experimental control and are, thus, critical to establish causality. Understanding is not gained by observation but by manipulation, physically, genetically, pharmacologically or immunologically, in ways that would be unreasonable or even unethical to do in a human.

Animal models are critical for investigating disease mechanisms. Diseases are exceptionally intricate, rarely involving a single phenotypic abnormality and often affecting multiple organ systems. A case in point is the multiple phenotypes observed in allergic disease. This presents a conundrum: on the one hand, deciphering this complexity in a human being is an indomitable task; on the other, animal models are a simplification of this reality and can only provide approximations. Consequently, the expectation that findings from a single model may wholly explain a heterogeneous disease is unrealistic. The implication is that multiple models asking multiple questions are necessary to provide a comprehensive view of a disease process, food allergy in this case. Animal models are extensively used as well to interrogate the biological impact of novel therapeutics. These studies along with other experimental approaches are critical to narrow the decision of which therapeutics should progress to large scale studies in humans. In other words, knowing what not-to-do helps immensely with the decision of what-to-do.

## Perspective on Food Allergy Modeling

Food allergy is typically diagnosed following a clinical reaction in infants or toddlers upon first known exposure (47). However, a prior event must have taken place to trigger allergic sensitization and, ultimately, production of food-specific IgE. As sensitization in humans is a silent process neither its initiation nor its evolution can be interrogated. Moreover, there appears to be no unifying genetic or environmental exposures, highlighting the heterogenous nature of food allergy. Accordingly, a diversity of animal models—primarily mouse models—have provided a tangible, ethical, and indispensable approach to decipher the cellular and molecular mechanisms underlying allergic sensitization.

Unlike some aeroallergens, toxins, and animal poisons, the vast majority of food antigens are not inherently immunogenic. As a consequence, most humans and experimental animals develop oral tolerance, an active state of non-reactivity, upon first exposure to food antigens (48, 49). In fact, the LEAP study demonstrated that over 98% of infants with atopic comorbidities (eczema and/or egg allergy) receiving their first exposure to peanut before 11 months of age develop oral tolerance (50). Similar to a type 2 homeostatic response, the tolerogenic response to food antigens [reviewed in citations (51, 52)] fundamentally involves DCs. However, acquisition of oral tolerance imperatively lacks the key molecules and the majority of cells that trigger Th2 polarization. It follows that a concomitant subversion of homeostasis is required to prime the immune system to mount a Th2-dominant response against food antigens (**Figure 1E**). In mouse modeling, this subversion is generally achieved through the use of adjuvants—substances which aid in eliciting robust immune responses (53). Cholera toxin (CT), staphylococcal enterotoxin B (SEB), and aluminum hydroxide (alum), are three of the most widely used biological or chemical adjuvants that facilitate a Th2-dominant response in mice (54–58). Damaged skin, due to physical disruption of epidermal barriers (i.e., epicutaneous sensitization), drives sensitization to topically applied allergen (59–63). A second approach to modeling food allergies, also a subversion of homeostasis, involves reducing the threshold of reactivity *via* genetic mutations. For example, *Il4ra^F709^* mice which contain a loss-of-function mutation in the *Il4ra* immunotyrosine inhibitory motif, display a lower sensitization threshold upon food allergen exposure (64, 65). Thus, the use of adjuvanted models has played a key role in overcoming the elusiveness of allergic sensitization in humans.

Basic discoveries that discriminate tolerant (homeostatic) *versus* pathogenic (allergic) responses to foods have been described using models in which mice are sensitized with a single or repeated administration of CT along with a food antigen (56, 66–68). CT is classified as an AB_5_ toxin; this class of toxins are produced by many enterotropic bacteria, such as *Escherichia coli* and *Bordetella pertussis* (69). Oral administration of CT enhances CD103^+^ CD11c^+^ DC migration to the mesenteric lymph nodes as well as expression of MHC II and costimulatory molecules (e.g., CD86) (70–72). These effects *per se* do not rationalize the elicitation of dominant Th2 priming. However, studies investigating the gene expression profile of mesenteric lymph node DCs in CT-immunized mice showed an upregulation of OX40L (70). Antibody-mediated blockade and genetic knockouts demonstrated a critical role of OX40L to promote Th2 skewing by enabling the initial burst of IL-4 from CD4 T cells (28, 41, 70). Further studies exposed the involvement of the IL-33/ST2 innate axis, where IL-33-signaling facilitates OX40L upregulation (41). Intriguingly, use of an ILC2 depletion strategy revealed a dispensable role of ILC2s in oral sensitization to foods, despite a dominant presence in intestinal tissues and a well-defined role in type 2 homeostatic processes (41).

A relationship between skin atopic abnormalities and sensitization to food allergens has become clear through large-scale longitudinal studies (73, 74). A primary example of this are loss-of-function mutations in the filaggrin gene (*Flg*) that correlate with peanut allergy in humans (75). To elucidate the immunological mechanisms of this relationship, models of epicutaneous sensitization have been employed. Sensitization is typically achieved through tape stripping, which removes the outermost epidermal layer, causing skin inflammation (63). These models have been referred to as “adjuvant-free”, which is misleading as tape stripping provides the same function as an adjuvant—induction of an immune response *via* an external input (76–78). Animal models have revealed an essential role of epithelial cells pertaining to barrier function and alarmin production during epicutaneous allergen exposure. In mice, disturbed production of filaggrin, a protein involved in epidermal barrier function, facilitated allergic sensitization to allergen applied topically (79). Damaged epithelial cells are an important source of alarmins such as IL-33, TSLP, and uric acid which, as reviewed earlier, enable DC activation and subsequent CD4 T cell priming (80–81). Tape-strip models have also revealed a role of ILC2s which, in response to IL-25 and IL-33 secretion from tape-stripped skin, proliferate in intestinal tissues to drive IL-4-dependent mast cell expansion (82).

In addition to elucidating the innate immunological events underlying allergic sensitization, animal modeling has enabled discoveries pertaining to allergic reactions. Alarmins involved in sensitization also participate in the effector phase. IL-33 interacts directly with mast cells to potentiate mast cell degranulation (84). Moreover, FcεRI-mediated mast cell activation stimulates production of IL-25, which is hypothesized to further drive the Th2 phenotype (85). Mouse models have elucidated the vasoactive mediators that facilitate anaphylaxis. In humans a correlation between anaphylactic severity and serum platelet activating factor (PAF) levels, but not histamine levels, was reported (86). Use of mouse models determined that blockade of histamine receptors (H1 and H2) was insufficient at preventing anaphylaxis, but that concurrent treatment with antihistamines and a PAF-receptor antagonist could significantly reduce anaphylactic severity (87).

In mouse models, allergen challenges vary greatly in dose and route (intraperitoneal, oral, intradermal, and intravenous) (56, 58, 88, 89). Routes of challenge differ in the clinical phenotype that is induced. For example, oral challenges tend to result in diarrhea and, sometimes, a mild drop in core body temperature. Intraperitoneal challenges, on the other hand, facilitate a much more robust drop in core body temperature and other severe systemic reactions (e.g., seizures). The severity of clinical reactivity may, in part, be dependent on antigen availability and mast cell density at the site of allergen administration (90). As well, IgG has also been shown to mediate anaphylaxis in food allergy mouse models, although an equivalent IgG-mediated food-induced anaphylactic pathway in humans remains contentious (63, 91, 92). In all instances—independent of allergen administration route—modeling allergen challenge reveals downstream functions of allergen-specific immunoglobulins (e.g., the events following allergen cross-linking of mast cell-bound IgE). Thus, the selection of the route of allergen challenge should be guided by the research question, where intraperitoneal and intravenous challenges are best suited to assess systemic anaphylaxis, oral challenge to evaluate gut-local effects (e.g., allergic diarrhea, goblet cell hyperplasia), and intradermal challenge to measure localized vascular permeability. Use of anti-FcγRII/III blocking antibodies may also be employed to further discriminate between IgE- and IgG-mediated allergen reactivity (91).

The use of animal models of food allergy for translational research has been scrutinized due to the necessity of “artificial/experimental sensitization” (i.e., requiring eccentric interventions). The validity of animal models for translational research can, however, be evaluated by three criteria: 1) face validity, 2) predictive validity, and 3) target validity (93, 94). Face validity evaluates the similarity of disease biology and symptomology between humans and the animal model (93). The biological processes of food allergy at large are conserved between mice and humans. For example, the immune response is dominantly Th2 polarized, IL-33 and uric acid are elevated compared to non-allergic controls, and clinical reactivity is IgE-mediated (84, 92, 95). Many of the clinical signs of an allergic reaction are also alike including itching, diarrhea, local inflammation, and systemic shock (76, 96). Predictive validity compares the effectiveness of interventions in humans and in the relevant animal model (93). Due to the small number of interventions tried in human food allergy, evaluation of predictive validity is limited, but oral immunotherapy provides a key example. Oral immunotherapy in both mice and humans can increase the threshold of reactivity (i.e., desensitization) to food allergens, although this desensitized state is, just as it is in the majority of humans, unsustained (68, 97). This limited assessment of predictive validity will be greatly improved come the initiation of clinical trials testing novel biotherapeutics (e.g., anti-IL-4Rα). Lastly, target validity establishes that a particular target (e.g., a cytokine) has the same function in humans and the disease model (93). Many of the molecules and cells involved in human food allergy provide similar actions in animal models. For instance, IL-4 promotes Th2 polarization and IgE class-switch in both mice and human food allergy (95). In contrast, the role of IgG in human food-induced anaphylaxis remains contentious and, therefore, mouse models would provide low target validity for investigation of IgG-targeted interventions. In summary, despite not knowing the “adjuvants” at play in human food allergy, adjuvant-based experimental models have revealed cellular and molecular signatures that exist in humans. Moreover, regardless of experimental model, it is clear that an innate program drives allergic sensitization.

## Concluding Remarks

Over the last few decades, there have been fundamental advances in our understanding of allergic disease, including food allergy. These advances have led to a dramatic re-conceptualization of the functions of type 2 immunity and the critical role of the innate system in programming these functions. We must recognize, however, that the current state of knowledge is vastly incomplete. For example, how gut dysbiosis stresses local tissue environments and impacts primary and secondary immune responses to foods is an emerging area already providing novel insights (98, 99). We surmise that experimental models will continue providing seminal discoveries on the machinery (cells and molecules) and the engineering (operating mechanisms) that underlie allergy. Indeed, the ability to overexpress or repress individual genes in animals, either permanently or on-demand, pervasively or in specific cell types, unlocks unique opportunities to decipher both the pathogenesis and the trajectory of the allergic diathesis. Lastly, experimental modeling remains an unparalleled sieve to guide what should be investigated in humans.

The ever-increasing application of technologies that deliver formidable datasets will help to delineate complex inter- and intracellular pathways, thus enlightening the astounding phenotypic diversity and plasticity of the allergic response. Mass cytometry has dramatically expanded upon flow cytometry in the analysis of protein expression at the single cell level and is drastically reshaping histology allowing for the measurement of very many markers on a single section. Bulk and single cell RNA sequencing is rapidly expanding in read depth, resulting in greater appreciation of the genetic signatures and subpopulations that define disease states. Constant advances in bioinformatics and machine learning continue to unveil new ways of mining these massive datasets for pathways and cell differentiation trajectories. There is no doubt that the next generation of large data analysis is already being conceived and developed beyond the gaze of immunologists. The logic of applying these analyses to human samples is clear. However, applying these technologies to animal research will remain imperative in at least two areas that are not currently possible in humans: 1) To address questions that require manipulation (genotype and exposure), not only observation, to understand how the system reacts and 2) To efficiently deconvolute the temporal and spatial evolution of a given perturbation or process; particularly those that are obscured by the complex exposure history of humans. Furthermore, emerging discoveries from these datasets in both humans and models will have to be validated through mechanistic studies in experimental models using the long-established immunological toolkit to decipher causal relationships.

## Author Contributions

KB, JK, AP, and MJ wrote and edited the manuscript. All authors contributed to the article and approved the submitted version.

## Funding

This work was supported by funds from Food Allergy Canada, Walter and Maria Schroeder Foundation, Michael Zych Family, and Canadian Asthma, Allergy, and Immunology Foundation (CAAIF). KB holds a Canada Graduate Scholarship. AP holds The Eva Eugenia Lillian Cope Scholarship.

## Conflict of Interest

The authors declare that the research was conducted in the absence of any commercial or financial relationships that could be construed as a potential conflict of interest.
